# Thalamic Structural Connectivity Alterations in Essential Tremor Associated with REM Sleep Behaviour Disorder

**DOI:** 10.5334/tohm.1088

**Published:** 2025-11-18

**Authors:** Elisa Bortolin, Camilla Calomino, Rita Nisticò, Giulia Bruschi, Maria V. Corbari, Andrea Quattrone, Aldo Quattrone, Fabiana Novellino, Maria Salsone

**Affiliations:** 1 Vita-Salute San Raffaele University, Milan, Italy; 2 Neuroscience Research Center, Department of Medical and Surgical Science, Magna Grecia University, Catanzaro, Italy; 3 Institute of Neurology, Department of Medical and Surgical Sciences, Magna Graecia University, Catanzaro, Italy; 4 IRCSS Policlinico San Donato, San Donato Milanese, Italy

**Keywords:** Essential Tremor, REM Sleep Behavior Disorder, structural connectivity, thalamus

## Abstract

**Background:**

REM sleep behavior disorder (RBD) is a rare REM-parasomnia, now considered a non-motor symptom of Essential Tremor (ET). Distinct structural alterations in the thalamus, as a key region modulating REM sleep, have been reported in patients with idiopathic and Parkinson’s disease-related forms. In this work, we investigated thalamic regions in ET patients with and without RBD, using a graph theoretical analysis.

**Methods:**

MRI data were acquired from 96 participants (41 ET, 10 ET with polysomnographic-confirmed RBD, ET-RBD, 45 controls). T1-weighted scans were obtained, and grey matter volumes were estimated across 28 thalamic regions of the AAL3 template (Cat12 toolbox). An adjacency matrix for each group was calculated using Pearson correlation. Group-specific matrices were extracted and nodal measures such as centrality measures and clustering coefficient were calculated. Differences between ET groups were computed using a set of 10000 random networks.

**Results:**

Interestingly, among analyzed thalamic regions, ET-RBD patients showed increased local strength and weighted clustering coefficient in Geniculate Body and increased Betweenness centrality in Right Pulvinar Inferior Nucleus (p = 0.05 FDR-corrected). Moreover, ET-RBD patients showed an increased strength and weighted clustering coefficient in Left Lateral Geniculate Body and Right Medial Geniculate Body, compared to controls (p = 0.05 FDR-corrected).

**Discussion:**

Our study demonstrates, for the first time, that the presence of RBD in ET is associated with an altered structural connectivity in thalamic regions. Our findings support the pathophysiologic role of the thalamus in the complex circuit causing RBD, in this particular ET phenotype.

**Highlights:**

- ET-RBD phenotype is associated with thalamic volume loss and altered structural connectivity, particularly in the medial and lateral geniculate and pulvinar nuclei.
- Our findings support the pathophysiologic role of the thalamus in the complex RBD pathophysiology in this particular ET phenotype.

## 1. Introduction

Essential tremor (ET) is one of the most common neurological diseases among adults. Clinically, it is a phenotypically heterogeneous disease characterized by both motor symptoms, including kinetic/postural tremor affecting the hand, head, and non motor symptoms such as REM sleep behavior disorder (RBD) [1,2,3]

RBD is a rare REM-parasomnia characterized by dream-enacting behaviors and loss of atonia during REM sleep [4]. The prevalence of RBD in ET is estimated to range between 8.7% and 18.5% [5], although this variability is strongly related to the methodology of assessment, questionnaire and/or polysomnography-based confirmation, and thus remains a topic of debate in the current literature. Importantly, it is well-documented that this prevalence is significantly higher than that found in the general aged population (ranging from 0.26 to 1.6%) [6,7,8,9]. Despite these findings, the clinical significance and underlying mechanisms of RBD in ET remain unclear. Notably, some authors propose that the presence of RBD in ET may reflect a pathophysiological link between ET and Parkinson’s Disease (PD) [5]. Indeed, RBD is now considered the most specific biomarker of phenoconversion of synucleopathies such as Parkinson’s Disease (PD) [10], thus its presence in the clinical spectrum of ET may corroborate the hypothesis that these two disorders are strictly related [11]. Interestingly, the presence of RBD within the ET clinical spectrum is often associated with a more complex phenotype, including additional biomarkers of phenoconversions such as hyposmia, autonomic dysfunctions, and cognitive impairments [3], further complicating its interpretation.

Neuroimaging studies have contributed to our understanding of the underlying mechanisms of PD and RBD pathogenesis. Previous reports were mainly focused on the PD-related and isolated forms of RBD. In detail, MRI structural differences in the thalamus, as a crucial structure involved in the sleep-wake cycle, have been reported in patients with PD associated with RBD, when compared to PD patients without RBD [12]. Similarly, in patients with the isolated form of RBD, a reduction of thalamic volume has been detected according to the cognitive status [13].

Given the limited understanding of RBD nature in ET, it remains to be determined whether its presence indicates a prodromal state, similar to PD-related RBD, or a specific underlying mechanism unique to ET. Understanding the nature of this distinction is crucial both for clarifying ET pathophysiology and for identifying potential biomarkers that distinguish RBD in ET from PD-associated RBD. This underscores the need to identify novel biomarkers of neurodegenerative RBD-related processes and networks. Neuroimaging provides a promising approach to investigate these mechanisms and uncover both disease-specific alterations and potential overlaps in pathological patterns.

In recent years, novel neuroimaging techniques have been developed. It is the case of the structural connectivity, the graph theoretical analysis. Centrality measures in graph theory are used to determine the importance or influence of a node within a network. In the context of brain networks, these measures help identify key brain regions (nodes) that play a critical role in organizing and integrating information across the brain. Graph theory provides a robust mathematical framework for examining the topological features of brain networks [14]. By representing the brain as a graph, it becomes possible to analyze the architectural organization of neural systems. Indeed, modelling data as networks, with nodes and edges, can reveal complex dependencies and community structures. Centrality and connectivity measures are especially useful in detecting influential nodes or clusters linked to minority classes. Thus, graph analysis offers two main advantages: it complements traditional classification by providing a different perspective and it helps address the challenges of class imbalance by focusing on relational information rather than class frequency alone.

To the best of our knowledge, there are no structural and/or functional neuroimaging studies investigating ET patients with RBD. In this study, we hypothesized that the thalamic involvement might be different across groups of patients. In particular, ET patients with RBD could also have thalamic alterations in several key regions modulating REM sleep. Thus, the purpose of this study was to explore the cortical–thalamic nuclei network based on volumetric data, employing graph theory to clarify how the structural characteristics of various thalamic nuclei relate to the cortex.

## 2. Materials and Methods

### 2.1 Participants

Forty-one patients diagnosed clinically with ET according to established criteria [15], 10 patients with ET and polysomnographically confirmed diagnosis of RBD based on the International Classification of Sleep Disorders, version 3 (ICSD-3) criteria [16], and 45 healthy controls were enrolled in the present study.

Each patient underwent a thorough clinical history review and a comprehensive neurological examination.

Particular importance was placed on gathering family history for tremor and PD. Family history was considered positive when a first-degree relative was reported to be affected. The Fahn-Tolosa scale was employed to clinically assess ET patients. Moreover, the symmetry of tremor or any asymmetry were evaluated. A comprehensive evaluation of motor symptoms was conducted with the motor section of the Movement Disorder Society-sponsored revision of the Unified Parkinson’s Disease Rating Scale (MDS-UPDRS) [17], and the olfactory function was assessed using the Sniffin’ Sticks test [18]. The clinical diagnosis of ET was supported by the integrity of nigrostriatal system on DAT-SPECT imaging, performed using a previously described protocol [19]. Briefly, after intravenous administration of 200 MBq of [^123I]FP-CIT, brain images were acquired 3 hours post-injection using a dual-headed SPECT camera (Infinia Hawkeye, General Electric, Milwaukee, WI). The images were reconstructed to perform qualitative and semiquantitative evaluation. Fixed-size regions of interest were bilaterally drawn over the striatum (bilateral caudate and putamen nuclei) and the occipital cortex, serving as the reference region. Specific-to-nonspecific binding was evaluated through ratios between specific uptake in bilateral striatal ROIs and mean occipital ROI uptake.

In all enrolled subjects, cognitive functions were evaluated and categorized as follows: i) global cognitive status (Mini Mental State Examination [MMSE]) [20], ii) executive functions (Frontal Assessment Battery [FAB] [21], Modified Card Sorting Test [MCST]) [22], Weigl’s Sorting Test [WEIGL] [23], iii) attention (Digit Span Forward) [24]; iv) verbal short- and long-term episodic memory (Rey Auditory-Verbal Learning Test Immediate [RAVLT-I] and Delayed [RAVLT-D]) [25]; v) visuospatial abilities (Judgments of Line Orientation test form V [JLO-V]) [26]; vi) language comprehension (Token Test) [27]; and vii) depressive symptoms (Beck Depression Inventory II [BDI-II]) [28]. Healthy controls were selected based on no history of neurological or significant general medical conditions and absence of vascular lesions on MRI scans. Written informed consent was obtained from all participants, and the study protocol was approved by the Ethical Committee of the University “Magna Graecia” of Catanzaro in accordance with the Helsinki Declaration.

### 2.2 MRI scanning and image processing

**Figure 1:**
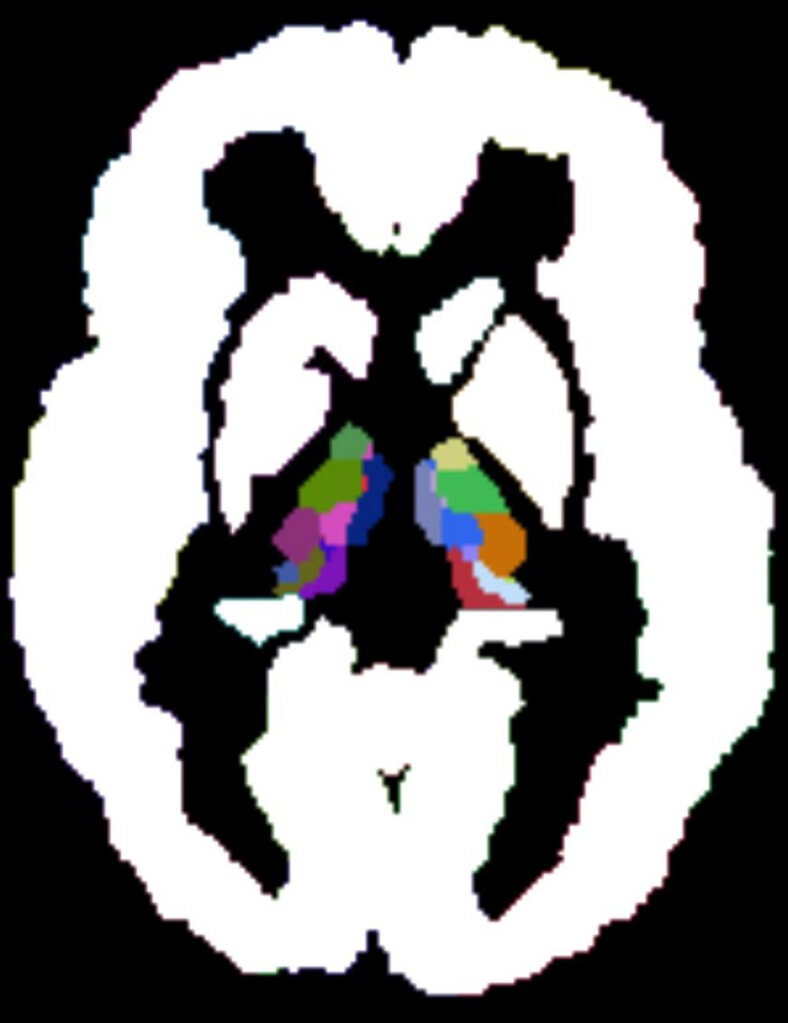
**Segmentation of the thalamic nuclei**. Automated segmentation of thalamic nuclei based on structural MRI data following aal3 atlas.

MRI brain scans were acquired from all participants using a 3 Tesla scanner equipped with an 8-channel head coil (Discovery MR-750, General Electric, Milwaukee, WI). To minimize head motion, foam pads were placed around participants’ heads. The MRI protocol consisted of a whole-brain T1-weighted scan [SPGR; Echo Time (ET) 3.7 ms, Repetition Time (TR) 9.2 ms, flip angle 12°, voxel size 1.0 × 1.0 × 1.0 mm^3^]. Structural T1-weighted images were processed using the Computational Anatomy Toolbox (CAT12) within SPM12 (www.fil.ion.ucl.ac.uk), running in the MATLAB environment (www.mathworks.com). Each participant’s images underwent normalization, starting with an affine registration followed by nonlinear registration, and were then corrected for bias field inhomogeneities. The images were subsequently segmented into gray matter (GM), white matter (WM), and cerebrospinal fluid (CSF) compartments. The DARTEL algorithm (Diffeomorphic Anatomic Registration Through Exponentiated Lie algebra algorithm) [29] was applied to warp the segmented scans into the standard Montreal Neurological Institute space. Modulation was performed to adjust for individual differences in brain size by applying nonlinear deformation to the normalized segmented images. Volumetric values were extracted based on the AAL3 template [30], selected due to its detailed subdivisions of the thalamus, which are critical regions for the disorders under investigation. Although the AAL3 template includes 166 cortical and subcortical regions, only 28 ROIs corresponding to thalamic subdivisions were included in the analysis, as shown in Figure 1, excluding the reuniens nucleus, which is considered too small to be reliably identified by typical registration processes in most individual brains.

### 2.3 Structural Covariance Networks Construction

The extracted volumes, as normalized fractions of the total intracranial volume (TIV), were used to construct gray matter structural correlation networks. An adjacency matrix representing the structural covariance network was generated for each group. In this framework, nodes corresponded to the selected 28 brain regions from the AAL3 template, and edges were defined as the pairwise Pearson correlation coefficients between the volumes of each region pair. Through this procedure, we obtained three symmetrical matrices (weighted 28 × 28), one per group. All negative correlations were set to zero.

### 2.4 Global and Local Network Metrics

NetworkX, a Python library, was employed for network analysis. At the global scale, the human connectome is typically characterized by a small-world topology. The small-worldness parameter (σ) is a scalar metric that incorporates two key properties: the clustering coefficient (C) and the characteristic path length (L) [31]. The clustering coefficient quantifies segregation by measuring the proportion of a node’s neighbors that are also connected to each other, while the characteristic path length reflects integration by representing the average shortest path between all pairs of nodes in the network. Both C and L are benchmarked against randomly generated null models, from which the normalized clustering coefficient (γ) and normalized path length (λ) are derived by comparing C and L to the average clustering coefficient (Crand) and path length (Lrand) of 10,000 randomized networks. The small-world index is then calculated as σ = γ/λ [32]. Networks with σ significantly greater than 1 are classified as exhibiting “small-world” properties. At the regional level, three graph metrics were computed and considered [33] (i) centrality measures such as connection strength and betweenness centrality; and (ii) segregation measures, including the weighted clustering coefficient. Nodal strength is defined as the sum of the weights of all edges connecting a node to others in the network, with higher values indicating greater connectivity to other brain regions. Nodal Betweenness Centrality (BC) represents the fraction of all shortest paths in the network that pass through a specific node. Elevated betweenness centrality indicates that a brain region plays a crucial role in facilitating information transfer within the network [34]. Conversely, reduced nodal strength and betweenness centrality in a particular brain region may suggest diminished communication efficiency and impaired information processing with the rest of the brain. To assess the segregation capacity of each region, the nodal clustering coefficient was also calculated, which reflects the proportion of a node’s neighbors that are interconnected, representing the node’s ability to engage in local communication. In this study, the weighted clustering coefficient was computed as the geometric mean of all triangles involving each node and, for the node i, it is defined as [35] \[
{{c}_{u}}\ =\ \frac{1}{deg(u)\left(deg(u)\ -\ 1\right))}\underset{vw}{\sum}\,{{\left({{\hat{w}}_{uv}}{{\hat{w}}_{uw}}{{\hat{w}}_{vw}}\right)}^{1/3}}
\] .

### 2.5 Comparing Network Metrics Between the Groups

Differences in nodal metrics were assessed between each pair of groups: (i) HCs vs ET patients; (ii) HCs vs ET-RBD patients; and (iii) ET vs ET-RBD patients. For each group comparison, inter-group differences in nodal network measures were evaluated using a non-parametric permutation method (10000 permutations). During each permutation, pairs of random networks preserving the original degree distribution [36] were created and used to compute the nodal metrics (strength, clustering coefficient, and betweenness centrality). This process generated a null distribution of differences for each node. The observed inter-group difference for each metric was then compared to the respective null distribution, and a p-value was derived for each brain region, defined as the proportion of permuted differences greater than the actual observed difference. To account for multiple comparisons, a false discovery rate correction was applied.

### 2.6 Statistical Analysis

Statistical analyses were conducted using Python. Mean values and standard deviation of demographic and clinical variables were calculated. We assessed the normality of data distribution using the Shapiro–Wilk test. To test whether the sample was well matched, the following statistical tests were performed: (i) ANOVA test including the three groups for age, education, MMSE, WEIGL, RAVLT-I, RAVLT-D, Digit Span forward, Token Test, Judgments of Line Orientation test form, Modified Card Sorting Test, Frontal Assessment Battery, Beck Depression Inventory II, followed by post hoc analysis with Bonferroni corrections, (ii) a two-sample t-test including ET and ET-RBD groups for disease duration, disease onset, Fahn Tolosa Scale (Total Score), MDS-UPDRS, left and right DAT-SPECT uptake (iii) a Fisher’s exact test for gender distribution, PD familiarity, tremor familiarity, postural asymmetry and postural side, hyposmia. Furthermore, ANCOVA with estimated total intracranial volume (ICV) as covariates was used to compare volumetric MRI data between groups. Spearman correlation analyses were conducted to assess the relationship between imaging measures and cognitive test scores in the entire ET cohort, including intracranial volume (ICV) as a covariate. Correlations between thalamic nuclei volumes and DAT-SPECT uptake values were also explored. In all statistical analyses, a p < 0.05 was considered significant after Bonferroni correction.

## 3. Results

### 3.1 Participants

The three groups were matched for gender distribution, age, and education. No statistical differences between the two groups of patients were found in terms of disease onset and duration, and Fahn-Tolosa and MDS-UPDRS part III scales. Family history of tremor and hyposmia were not different between ET patients with and without RBD. Tremor asymmetry was more common among ET-RBD patients, reaching a trend that bordered on statistical significance (p = 0.078). Regarding cognitive scores, no significant differences were found in the comparison between the three groups, nor in the post-hoc between ET and ET-RBD, with the exception of the Mini-Mental State Examination, where ET patients had a MMSE score significantly lower than HC (p = 0.008). A trend close to significance was found in RAVLT-I, in which ET-RBD patients performed worse (p = 0.060). The DAT-SPECT uptake showed no significant differences between ET with and without RBD groups bilaterally. More details are reported in Table 1.

**Table 1:** Demographic and clinical data of patients with ET stratified according to the presence or absence of RBD, and HC.


DATA	ET (N = 41)	ET-RBD (N = 10)	HC (N = 45)	p-value

Gender, (M/F)	19/22	7/3	22/23	0.398^a^

Age at examination, ys^b^	65.7 ± 10.0	62.9 ± 12.1	65.9 ± 9.48	0.454^c^

Education, ys^b^	9.1 ± 4.9	10.8 ± 2.5	12.2 ± 4.4	0.094^c^

Age at disease onset, ys^b^	50.6 ± 17.8	55.0 ± 11.9	–	0.430^d^

Disease duration, ys^b^	15.8 ± 16.2	7.9 ± 3.4	–	0.135^d^

PD familiarity (Y/N)	5/36	1/9	–	0.847^a^

Tremor familiarity	25/16	4/6	–	0.197^a^

Postural asimmetry	25/16	7/3	–	0.078^a^

Postural side (BL/DX/SX)	16/17/8	7/2/1	–	0.398^a^

Hyposmia (Y/N)	3/38	1/9	–	0.777^a^

Fahn-Tolosa^b^	23.0 ± 13.6	18.2 ± 7.3	–	0.329^d^

MDS-UPDRS part III^b^	5.1 ± 1.7	5.15 ± 1.82	–	0.940^d^

MMSE^b^	25.4 ± 4.0	26.7 ± 2.7	28.7 ± 1.7	**0.021**^c^,*

WEIGL^b^	11.2 ± 2.9	8.4 ± 2.1	10.1 ± 2.8	0.352^c^

RAVLT-I^b^	37.4 ± 10.9	31.5 ± 8.3	39.9 ± 7.1	0.210^c^

RAVLT-R^b^	7.4 ± 2.8	4.8 ± 2.3	6.8 ± 2.1	0.060^c^

DIGIT-S-F^b^	5.5 ± 3.7	4.5 ± 4.2	5.3 ± 4.0	0.882^c^

Token Test^b^	30.1 ± 2.6	29.1 ± 2.3	30.9 ± 1.65	0.263^c^

JLO^b^	22.2 ± 5.8	19.7 ± 5.1	22.8 ± 4.79	0.676^c^

MCST^b^	4.8 ± 2.3	4.7 ± 2.3	5.21 ± 1.31	0.809^c^

FAB^b^	14.1 ± 2.1	14.3 ± 1.2	15.2 ± 2.04	0.288^c^

BDI-II^b^	10.6 ± 5.6	14.0 ± 5.3	8.5 ± 5.03	0.314^c^

DAT-SPECT right (putamen/occipital cortex ratio)^b^	4.4 ± 0.6	4.6 ± 0.5	–	0.374^d^

DAT-SPECT left (putamen/occipital cortex ratio)^b^	4.4 ± 0.6	4.5 ± 0.6	–	0.431^d^


Abbreviations: ET = Essential Tremor; ET-RBD = Essential tremor with Rem Sleep Behavior Disorders; HC = Healthy controls; MMSE = Mini Mental State Examination; RAVLT-I = Rey Auditory-Verbal Learning Test Immediate; RAVLT-D = Rey Auditory-Verbal Learning Test Delayed; DIGIT-S-F = Digit Span Forward; JLO = Judgment of Line Orientation; MCST = Modified Card Sorting Test; FAB = Frontal Assessment Battery; BDI-II = Beck Depression Inventory II; WEIGL = Weigl’s Sorting test. ^a^Fisher’s exact test. ^b^Data are expressed as mean ± standard deviation. ^c^ANOVA test; ^d^t-test with Bonferroni correction. Significant p values are in bold. *post-hoc: ET< HC, p = 0.008.

### 3.2 Thalamic volume

Linear analysis on thalamic nuclei showed in ET with RBD patients a slight volume decrease of the bilateral Ventral Anterior Thalamic Nuclei in comparison with ET and HC with Bonferroni correction (Left: ET: 0.056 ± 0.01; ET-RBD: 0.044 ± 0.02; HC: 0.0613 ± 0.02; p = 0.047; Right: ET: 0.049 ± 0.01; ET-RBD: 0.039 ± 0.02; HC: 0.050 ± 0.02; p = 0.042). Detailed volumes of all thalamic nuclei and statistical significance are reported in Supplementary Table 1. Correlation analysis in the entire cohort of ET highlithed a moderate correlation between different thalamic nuclei and cognitive test. In detail, Left Anterior Thalamic Nucleus showed a correlation with MMSE (p = 0.009), DAT SCAN left (p < 0.001) and DAT SCAN right (p = 0.003). Moreover, the DAT SCAN correlated with Right Anterior Thalamic Nucleus and Left Mediodorsal Medial (p = 0.046 and p = 0.022 respectively), as showed in Table 2 and Supplementary Figure 1. After Bonferroni correction, only the correlation between the Left Anterior Thalamic Nucleus and the DAT SCAN left remains.

**Table 2:** Correlation between ROI of thalamic nuclei and variables in ET patients expressed as r-value and p-value between parenthesis.


ROI	CORRELATED TESTS r-value (p-value)			

Left Anterior Thalamic Nucleus	MMSE r = 0.38 (0.009)	FAB r = 0.33 (0.043)	DAT-SCAN right r = 0.42 (0.003)	DAT-SCAN left r = 0.46 (<0.001)

Right Anterior Thalamic Nucleus	MMSE r = 0.30 (0.044)	TOKEN r = 0.37 (0.027)	DAT-SCAN left r = 0.28 (0.046)	

Right Ventral Posterolateral Thalamic Nucleus	DIGIT-S-F r = –0.44 (0.020)			

Left Intralaminar Nucleus	MMSE r = 0.34 (0.021)			

Left Mediodorsal Medial Nucleus	MMSE r = 0.35 (0.017)	FAB r = 0.40 (0.015)	DAT-SCAN right r = 0.29 (0.044)	DAT-SCAN left r = 0.32 (0.022)

Right Mediodorsal Medial Nucleus	FAB r = 0.32 (0.050)			

Left Lateral Geniculate Body	WEIGL r = –0.63 (0.036)			

Right Lateral Geniculate Body	WEIGL r = –0.80 (0.003)			

Left Pulvinar Medial Nucleus	MMSE r = 0.36 (0.015)			

Right Pulvinar Medial Nucleus	MMSE r = 0.32 (0.034)			

Left Pulvinar Lateral Nucleus	MMSE r = 0.37 (0.011)	FAB r = 0.33 (0.032)		


Estimated total intracranial volume is used as covariate. There are showed only significant ROIs. MMSE = Mini Mental State Examination; FAB = Frontal Assessment Battery; DIGIT-S-F = Digit Span Forward; WEIGL = Weigl’s Sorting test.

**Figure 2:**
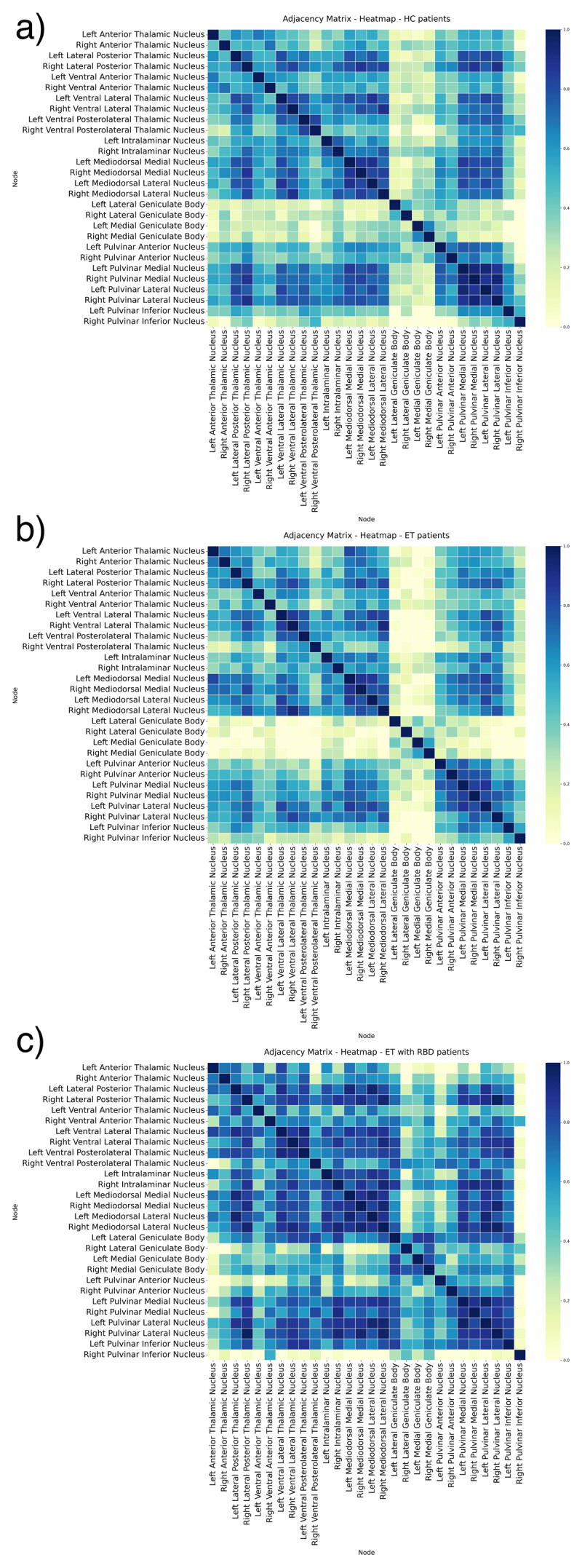
**Adjacency matrices of undirected graphs network in Healthy Controls (a), Essential Tremor (b) and Essential Tremor with RBD (c)**. The figures show the partial correlation with total intracranial volume as covariates, from each ROI. A higher number of connections among nodes is visible in the Essential Tremor with RBD compared to Healthy Control and Essential Tremor. The correlation strength is shown using a colour bar.

The gray matter structural correlation networks between ROIs are shown in Figure 2, with adjacency matrices (a, b, c). The the ET-RBD subjects exhibit a higher degree of connections among bilaterally homologous regions in comparison to what observed in HC and ET groups. On the contrary, the ET group had a pattern similar to HC.

### 3.3 Global network metrics

At a global level, the small-worldness index exceeded the value of 1 in all groups. The overall organization of the brain networks was preserved in all groups (Supplementary Table 2).

### 3.4 Local network metrics

#### 3.4.1 ET patients versus healthy controls

No significant difference survived between ET and HC. With an uncorrected approach, ET showed a lower betweenness centrality compared to HC in the Left Medial Geniculate Body, Left Pulvinar Inferior Nucleus, and Right Mediodorsal Lateral Nucleus. Moreover, ET showed a reduced Weighted clustering coefficient in the Left Medial Geniculate body. No differences were found in terms of local strength. Details are reported in Supplementary Table 3.

#### 3.4.2 ET-RBD patients versus healthy controls

**Figure 3:**
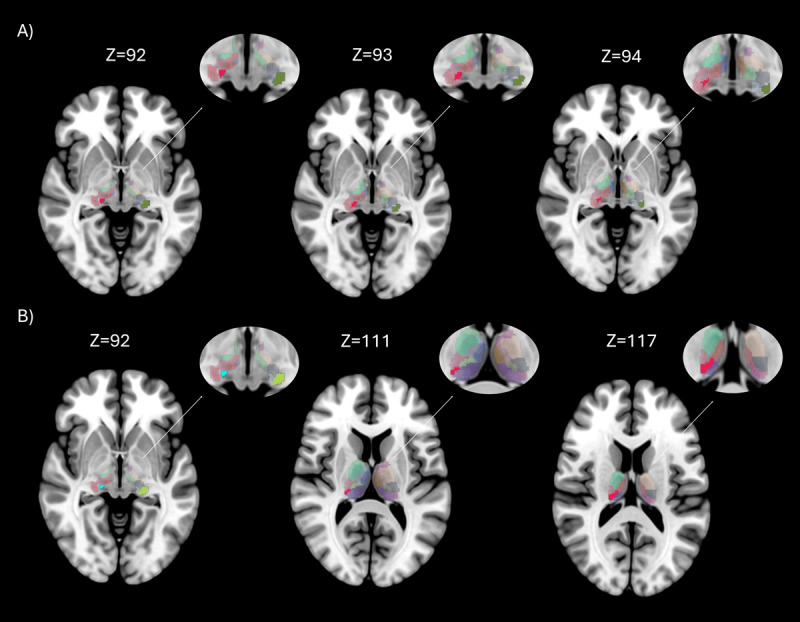
**Brain regions with significant differences in nodal properties between groups**. Rendering showing regions with significant differences between groups, in axial brain view. The entire thalamus with aal3 atlas is showed in background, while areas with statistically significant differences (p < 0.05 FDR corrected) are highlighted in different colors. Figure 3A shows the differences between Essential Tremor with RBD and Healthy Controls on different axial slices, in red is showed the Right Medial Geniculate Body and in green the Left Lateral Geniculate Body; Figure 3B between Essential Tremor (ET) and Essential Tremor with RBD (ET-RBD) on different axial slices, in light blue is showed the Right Medial Geniculate Body, in green the Left Lateral Geniculate Body, in grey the Right Medial Geniculate body and in red the Pulvinar Inferior.

As shown in Table 3 and Figure 3a, ET-RBD patients showed an increased strength in the Left Lateral Geniculate Body (p = 0.002) and Right Medial Geniculate Body (p = 0.014) compared to HC. In terms of the nodal weighted clustering coefficient, ET-RBD patients showed a higher clustering coefficient in the Left Lateral Geniculate Body than in HC (p = 0.004). No differences were found in terms of local betweenness centrality. Only FDR-corrected results are reported in the main text; uncorrected results are provided in Supplementary Table 4 for additional details.

**Table 3:** Brain regions contributing to group differences in the nodal analysis between ET-RBD and HC.


BRAIN REGION ET-RBD > HC	LOCAL GRAPH MEASURE	HC	ET-RBD	DIFFERENCE	p-VALUE

Left Lateral Geniculate Body	Strength	5.9	18.2	–12.3	0.002

Right Medial Geniculate Body	Strength	6.5	14.9	–8.4	0.014

Left Lateral Geniculate Body	Weighted clustering coefficient	0.29	0.60	–0.31	0.004


All the p-value are corrected with FDR method.

#### 3.4.3 ET versus ET-RBD patients

ET patients with RBD showed an increased local strength in the Left Lateral Geniculate Body (p = 0.006) and bilateral Medial Geniculate bodies (left: p = 0.007; right: p = 0.008). Moreover, compared to ET, ET-RBD showed a higher Betweenness centrality in the Right Pulvinar Inferior Nucleus (p = 0.038) (Table 4 and Figure 3b). Similarly, in terms of the Weighted clustering coefficient, ET-RBD increased in the Left Lateral Geniculate Body (p = 0.002) and bilateral Medial Geniculate Body (left: p = 0.007; right: p = 0.004). Only FDR-corrected results are reported in the main text; uncorrected tables are provided in the Supplementary Table 5 for additional details.

**Table 4:** Brain regions contributing to group differences in the nodal analysis between ET and ET-RBD.


BRAIN REGION ET-RBD > ET	LOCAL GRAPH MEASURE	ET	ET-RBD	DIFFERENCE	p-VALUE

Left Lateral Geniculate Body	Strength	3.89	18.2	–14.3	0.006

Left Medial Geniculate Body	Strength	2.51	12.6	–10.09	0.007

Right Medial Geniculate Body	Strength	3.34	14.9	–11.56	0.008

Right Pulvinar Inferior Nucleus	Betweenness centrality	0.23	0.64	–0.41	0.038

Left Lateral Geniculate Body	Weighted clustering coefficient	0.20	0.60	–0.40	0.002

Left Medial Geniculate Body	Weighted clustering coefficient	0.17	0.47	–0.30	0.007

Right Medial Geniculate Body	Weighted clustering coefficient	0.18	0.52	–0.34	0.004


All the p-value are corrected with FDR method.

## 4. Discussion

The current study provides evidence that the presence of RBD in patients with ET is associated with alterations in both volume and structural connectivity of thalamic regions, reinforcing the central role of the thalamus in the pathophysiology of this specific ET phenotype.

In particular, ET-RBD patients exhibited increased local connectivity in the Left Lateral Geniculate Body and the bilateral Medial Geniculate Bodies. These connectivity alterations align with the known involvement of sensory thalamic relay stations in REM sleep mechanisms. In particular, the Lateral Geniculate Body is the primary thalamic relay region for visual information and, durning REM sleep, it is actively involved in phasic activation and in visual imagery associated with dreaming [37]. On the other hand, the Medial Geniculate Body is a key auditory relay region [38] and has been shown in animal studies to transmit auditory signals during REM sleep [39]. Another relevant finding was the increased Betweenness Centrality in the Right Pulvinar Inferior Nucleus in ET-RBD compared to ET patients without RBD. The pulvinar serves as a higher-order visual relay station [38], playing a crucial role in visual processing and in connecting and integrating information across cortical areas [40]. Specifically, this nucleus has been implicated as a hub for multisensory integration during phasic REM sleep [41]. Recent research also highlights its role in maintaining thalamo-cortical communication. Accordingly, its disruptions may be linked to impaired spatio-temporal binding possibly underlying the altered perceptual experiences characteristic of dreams during REM sleep [42]. These results extend previous functional imaging studies showing thalamic degeneration in idiopathic RBD and PD patients with RBD, correlating with the severity of RBD symptoms [43]. Indeed, the thalamus has been consistently implicated in the pathogenesis of RBD [12], which is believed to originate from dysfunction in the neuronal circuits responsible for the physiological inhibition of muscle activity during REM sleep [44]. Cholinergic and monoaminergic brainstem neurons are central to this process, and their dysfunction has been repeatedly demonstrated in iRBD, with PET studies revealing metabolic alterations [45] and reduced cholinergic innervation in the thalamus [12], brainstem [46], and cortex [47].

Beyond connectivity changes, ET-RBD patients also showed a slight decrease in the volume of both Ventral Anterior Thalamic Nuclei compared to both ET patients without RBD and HC, consistent with previous reports of thalamic volume loss in PD patients with RBD versus those without [12]. Given the central role of these nuclei in motor planning and control, and their integration within basal ganglia-thalamocortical circuits [48], their involvement may contribute to the clinical presentation observed in ET-RBD.

This is the first study highlighting the specific contributions of the Geniculate Bodies and the Pulvinar to RBD pathophysiology, which remain still underexplored and warrant further investigation. Increased connectivity is a common finding in neurodegenerative diseases, including PD and DLB [49,50,51,52]. Connectivity changes are often understood in terms of function [53]: the increased connectivity of these nuclei in ET-RBD may reflect heightened functional integration within the thalamus in the context of reduced volume and progressive pathology, indicating a “beneficial” compensatory reorganization. This compensatory hypothesis is especially plausible when the observed enhanced connectivity is correlated with lower symptom severity [53]. However, in the literature, higher connectivity has also been associated with more severe clinical symptoms or faster disease progression, suggesting a pathogenic mechanism rather than a compensatory one [53]. In the present study, given the additional motor and sensory disturbances associated with thalamic volumes and connectivity alterations characterizing this subgroup, it may alternatively signal a maladaptive broader reorganization of thalamocortical integration mechanisms specific to the ET-RBD phenotype. Overall, our findings point to greater thalamic changes in ET-RBD compared to ET alone, potentially explaining the additional motor and sensory disturbances seen in ET-RBD and supporting its identification as a distinct neuroanatomical phenotype.

Neuropathological evidence reported the presence of Lewy bodies (LB) in a subset of ET patients [54], indicating that ET may represent a pathophysiologically heterogeneous condition, with epidemiological data showing a fourfold increased risk of developing PD among ET patients [55]. Therefore, one possible explanation is that ET-RBD subjects might represent a subset of patients with an incident early stage of PD. Supporting this hypothesis, neuropathological studies [56,57,58] have highlighted thalamic involvement in RBD, especially in the intralaminar [57] and limbic system nuclei [56], which are key regions in LB diseases and primary extranigral targets of Lewy pathology. Additionally, thalamic LB pathology appears more prominent in PD cases with RBD than those without sleep disturbances [58]. Consistent with this interpretation, a recent study reported that 60% of ET-RBD patients met criteria for prodromal PD, compared to only 1.5% of ET patients without RBD [5]. However, it is important to emphasize that this hypothesis remains debated. Not all pathological studies confirm a higher frequency of LB in ET patients, and some reports suggest that ET does not represent early PD, given the lack of striatal dopaminergic deficits compared to controls [59,60,61]. These findings suggest that ET-RBD may not uniformly reflect a prodromal synucleinopathy, but might instead represent a clinically and pathophysiologically heterogeneous group. An alternative view proposes that longstanding ET may somehow lead to LB formation, rather than resulting from them [60].

Regarding the characteristic of patients enrolled in the present study, all ET and ET-RBD patients had normal DAT-SCAN scintigraphy. Notably, anterior and mediodorsal thalamic nuclei volumes were significantly correlated with putaminal uptake values, indicating a relationship between thalamic structural integrity and dopaminergic pathway function. Furthermore, ET-RBD patients had a higher prevalence of tremor asymmetry, further demonstrating some similarity of this clinical phenotype to the clinical features of PD. Taken together, these findings underscore the complexity of interpreting RBD in ET. While thalamic alterations and clinical features overlap with PD, the current evidence does not conclusively determine whether ET-RBD patients represent prodromal PD or a distinct ET-related phenotype with unique pathophysiological mechanisms. Regardless of the different perspectives, our findings provide direct evidence of thalamic degeneration in ET-RBD, indicating a selective vulnerability of thalamic circuits that may underlie the clinical phenotype of this subgroup.

This study has some limitations. First of all, the small sample size, particularly of the ET-RBD group, which reflects the prevalence of RBD symptoms in ET patients, limits our ability to draw definitive conclusions. Additionally, a direct comparison with prodromal PD patients was not performed in this study. Such a comparison would be valuable in future research to clarify specific differences and overlaps between ET-RBD and prodromal PD, thereby better delineating the distinct pathophysiological mechanisms underlying these conditions.

Our work has several strengths. Firstly, the novelty. It is the first neuroimaging study investigating structural differences in patients with ET associated with RBD. Secondly, RBD has been PSG confirmed, thus we are confident that these symptoms are really manifested by our patients. Finally, graph-based network analysis is a valuable approach, exspecially when dealing with imbalanced groups.

In conclusion, our study demonstrates that the ET-RBD subset of patients shows thalamic alterations compared to ET without RBD, thus supporting the thalamus’s key role in the RBD genesis. To date, we don’t know whether this particular phenotype really belongs to ET pathology rather than will convert into Parkinsonian syndrome. Further cross-sectional and longitudinal studies conducted in a large cohort of ET-RBD patients are needed to confirm our results.

## Additional Files

The additional files for this article can be found as follows:

10.5334/tohm.1088.s1 Supplementary Tables. Supplementary Tables S1 to S5.

10.5334/tohm.1088.s2 Supplementary Figure. Significant correlation between Thalamic volumes and DAT-SCAN uptake in the full Essential Tremor cohort.
