# Supplementary material for: Thalamic Structural Connectivity Alterations in Essential Tremor Associated with REM Sleep Behaviour Disorder
Source: Tremor Other Hyperkinet Mov (N Y). 2025 Nov 18;15:58. doi: 10.5334/tohm.1088 (PMC12636279; doi:10.5334/tohm.1088)
Supplement: Supplementary Tables. — Supplementary Tables S1 to S5. [file tohm-15-1-1088-s1.pdf]

Supplementary Table 1. Volumes of thalamic regions of interest in patients with Essential Tremor with and without REM Sleep Behavior Disorder (RBD), and in healthy control subjects.

Data	ET (N=41)	ET-RBD (N=10)	HC (N=45)	p-value
Left Anterior Thalamic Nucleus[a]	0.048 ± 0.01	0.047 ± 0.01	0.049 ± 0.01	0.879[c]
Right Anterior Thalamic Nucleus[a]	0.073 ± 0. 01	0.071 ± 0. 02	0.072 ± 0. 01	0.963[c]
Left Lateral Posterior Thalamic Nucleus[a]	0.106 ± 0. 02	0.104 ± 0. 02	0.108 ± 0. 01	0.760[c]
Right Lateral Posterior Thalamic Nucleus	0.127 ± 0.02	0.117 ± 0.03	0.124 ± 0.02	0.460[c]
Left Ventral Anterior Thalamic Nucleus[a]	0.056 ± 0.01	0.044 ± 0.02[*]	0.0613 ± 0.02	**0.047[c]**
Right Ventral Anterior Thalamic Nucleus[a]	0.049 ± 0.01	0.039 ± 0.02[*]	0.050 ± 0.02	**0.042[c]**
Left Ventral Lateral Thalamic Nucleus[a]	0.730 ± 0.13	0.690 ± 0.19	0.750 ± 0.13	0.337[c]
Right Ventral Lateral Thalamic Nucleus[a]	0.663 ± 0.11	0.613 ± 0.17	0.660 ± 0.13	0.504[c]
Left Ventral Posterolateral Thalamic Nucleus[a]	0.443 ± 0.06	0.440 ± 0.09	0.450 ± 0.09	0.886[c]
Right Ventral Posterolateral Thalamic Nucleus[a]	0.297 ± 0.06	0.296 ± 0.06	0.291 ± 0.06	0.883[c]
Left Intralaminar Nucleus[a]	0.094 ± 0.02	0.101 ± 0.02	0.097 ± 0.02	0.456[c]

Data	ET (N=41)	ET-RBD (N=10)	HC (N=45)	p-value
Right Intralaminar Nucleus[a]	0.110 ± 0.02	0.110 ± 0.03	0.105 ± 0.02	0.661[c]
Left Mediodorsal Medial Nucleus[a]	0.651 ± 0.09	0.615 ± 0.12	0.658 ± 0.10	0.452[c]
Right Mediodorsal Medial Nucleus[a]	0.765 ± 0.10	0.726 ± 0.15	0.765 ± 0.12	0.607[c]
Left Mediodorsal Lateral Nucleus[a]	0.209 ± 0.04	0.188 ± 0.05	0.219 ± 0.04	0.077[c]
Right Mediodorsal Lateral Nucleus[a]	0.221 ± 0.04	0.206 ± 0.06	0.220 ± 0.05	0.625[c]
Left Lateral Geniculate Body[a]	0.038 ± 0.02	0.036 ± 0.01	0.037 ± 0.02	0.816[c]
Right Lateral Geniculate Body[a]	0.033 ± 0.01	0.037 ± 0.01	0.032 ± 0.01	0.462[c]
Left Medial Geniculate Body[a]	0.014 ± 0.01	0.010 ± 0.02	0.015 ± 0.02	0.133[c]
Right Medial Geniculate Body[a]	0.011 ± 0.004	0.012 ± 0.005	0.010 ± 0.003	0.612[c]
Left Pulvinar Anterior Nucleus[a]	0.082 ± 0.01	0.081 ± 0.01	0.081 ± 0.01	0.908[c]
Right Pulvinar Anterior Nucleus[a]	0.065 ± 0.01	0.069 ± 0.01	0.066 ± 0.01	0.630[c]
Left Pulvinar Medial Nucleus[a]	0.892 ± 0.12	0.886 ± 0.15	0.893 ± 0.15	0.991[c]

Data	ET (N=41)	ET-RBD (N=10)	HC (N=45)	p-value
Right Pulvinar Medial Nucleus[a]	0.949 ± 0.12	0.919 ± 0.20	0.929 ± 0.16	0.770[c]
Left Pulvinar Lateral Nucleus[a]	0.172 ± 0.02	0.165 ± 0.04	0.177 ± 0.03	0.443[c]
Right Pulvinar Lateral Nucleus[a]	0.174 ± 0.02	0.171 ± 0.03	0.173 ± 0.03	0.963[c]
Left Pulvinar Inferior Nucleus[a]	0.077 ± 0.01	0.072 ± 0.01	0.076 ± 0.01	0.557[c]
Right Pulvinar Inferior Nucleus[a]	0.051 ± 0.01	0.053 ± 0.01	0.051 ± 0.01	0.891[c]

.
[a]Data are expressed as mean ± standard deviation. [c]ANCOVA with TIV as covariate, followed by post hoc with Bonferroni correction.
*ET-RBD<HC

Supplementary Table 2. Global measures in essential tremor patients with and without RBD and healthy controls.

Global graph measures	ET	ET-RBD	HC
Density	0.95	0.87	0.97
Normalized Clustering Coefficient	1.57	1.54	1.57
Normalized Path Length	0.76	0.80	0.75
Small-worldness	2.05	1.93	2.08

Supplementary Table 3. Brain regions contributing to group differences in the nodal analysis between healthy controls and essential tremor.

**HC vs ET**<br><br>**Brain region**	**Local graph measure**	**HC**	**ET**	**Difference**	***p* value**<br><br>**uncorrected**	**Comparison**
Left Medial Geniculate Body	Betweenness	0.42	0.23	0.19	0.045	ET< HC
Left Pulvinar Inferior Nucleus	Betweenness	0.50	0.24	0.26	0.020	ET< HC
Right Mediodorsal Lateral Nucleus	Betweenness	0.39	0.20	0.19	0.025	ET< HC
Right Lateral Geniculate	Betweenness	0.60	0.28	0.32	0.035	ET< HC
Left Medial Geniculate	Weighted clustering coefficient	0.50	0.29	0.21	0.024	ET< HC

Supplementary Table 4. Brain regions contributing to group differences in the nodal analysis between healthy controls and essential tremor with RBD.

**HC vs ET-RBD**<br>**Brain region**	**Local graph measure**	**HC**	**ET-RBD**	**Difference**	***p* value**<br>**uncorrected**	**Comparison**
Left Lateral Geniculate Body	Strength	5.9	18.2	-12.3	<0.001	ET-RBD> HC
Left Medial Geniculate Body	Strength	6.5	12.63	-12.3	0.022	ET-RBD> HC
Left Pulvinar Inferior Nucleus	Strength	10.8	17.7	-12.3	0.006	ET-RBD> HC
Right Medial Geniculate Body	Strength	6.5	14.9	-8.4	<0.001	ET-RBD> HC
Right Anterior Thalamic Nucleus:	Betweenness	0.39	0.60	-0.21	0.047	ET-RBD> HC
Left Pulvinar Anterior Nucleus	Betweenness	0.48	0.61	-0.13	0.046	ET-RBD> HC
Right Pulvinar Medial Nucleus	Betweenness	0.35	0.62	-0.27	0.027	ET-RBD> HC
Right Pulvinar Inferior Nucleus	Betweenness	0.37	0.69	-0.32	0.033	ET-RBD> HC
Left Lateral Geniculate Body	Weighted clustering coefficient	0.29	0.60	−0.31	<0.001	ET-RBD> HC
Right Medial Geniculate Body	Weighted clustering coefficient	0.31	0.52	−0.21	0.003	ET-RBD> HC

Supplementary Table 5. Brain regions contributing to group differences in the nodal analysis between essential tremor with and without RBD.

ET vs ET-RBD<br>Brain region	Local graph measure	ET	ET-RBD	Difference	*p* value<br>uncorrected	Comparison
Left Lateral Geniculate Body	Strength	3.89	18.2	−14.3	<0.001	ET-RBD > ET
Left Medial Geniculate Body	Strength	2.51	12.6	-10.09	<0.001	ET-RBD > ET
Right Medial Geniculate Body	Strength	3.34	14.9	-11.56	<0.001	ET-RBD > ET
Left Anterior Thalamic Nucleus	Betweenness centrality	0.22	0.61	-0.39	0.039	ET-RBD > ET
Left Lateral Geniculate Body	Betweenness centrality	0.20	0.66	-0.46	0.043	ET-RBD > ET
Right Lateral Geniculate Body	Betweenness centrality	0.19	0.59	-0.40	0.028	ET-RBD > ET
Left Medial Geniculate Body	Betweenness centrality	0.27	0.64	-0.37	0.006	ET-RBD > ET
Right Medial Geniculate Body	Betweenness centrality	0.21	0.61	-0.40	0.042	ET-RBD > ET
Left Pulvinar Inferior Nucleus	Betweenness centrality	0.20	0.61	-0.41	0.040	ET-RBD > ET

**ET vs ET-RBD**<br>**Brain region**	**Local graph measure**	**ET**	**ET-RBD**	**Difference**	***p* value**<br>**uncorrected**	**Comparison**
Right Pulvinar Inferior Nucleus	Betweenness centrality	0.23	0.64	-0.41	<0.001	ET-RBD > ET
Left Lateral Geniculate Body	Weighted clustering coefficient	0.20	0.60	−0.40	<0.001	ET-RBD > ET
Left Medial Geniculate Body	Weighted clustering coefficient	0.17	0.47	−0.30	<0.001	ET-RBD > ET
Right Medial Geniculate Body	Weighted clustering coefficient	0.18	0.52	−0.34	<0.001	ET-RBD > ET
