# Supplementary material for: Thalamic Structural Connectivity Alterations in Essential Tremor Associated with REM Sleep Behaviour Disorder
Source: Tremor Other Hyperkinet Mov (N Y). 2025 Nov 18;15:58. doi: 10.5334/tohm.1088 (PMC12636279; doi:10.5334/tohm.1088)
Supplement: Supplementary Figure. — Significant correlation between Thalamic volumes and DAT-SCAN uptake in the full Essential Tremor cohort. [file tohm-15-1-1088-s2.pdf]

Legend to the Supplementary Figures:

Supplementary Figure 1: Significant correlation between Thalamic volumes and DAT-SCAN uptake in the full Essential Tremor cohort.

Scatter plots showing the correlation between thalamic nuclei volumes and striatal dopamine transporter uptake (DAT-SCAN), expressed as the putamen-to-occipital cortex ratio in Essential Tremor patients:

(A) Correlation between Left Anterior Thalamic Nucleus and right DAT-SPECT uptake.

(B) Correlation between Left Anterior Thalamic Nucleus and left DAT-SPECT uptake.

(C) Correlation between Right Anterior Thalamic Nucleus and left DAT-SPECT uptake.

(D) Correlation between Left Mediodorsal Medial Nucleus and right DAT-SPECT uptake.

(E) Correlation between Left Mediodorsal Medial Nucleus and left DAT-SPECT uptake.

Red lines represent the linear regression fit. Pearson's correlation coefficients (r) are shown in each panel. Each blue dot represents a single participant.

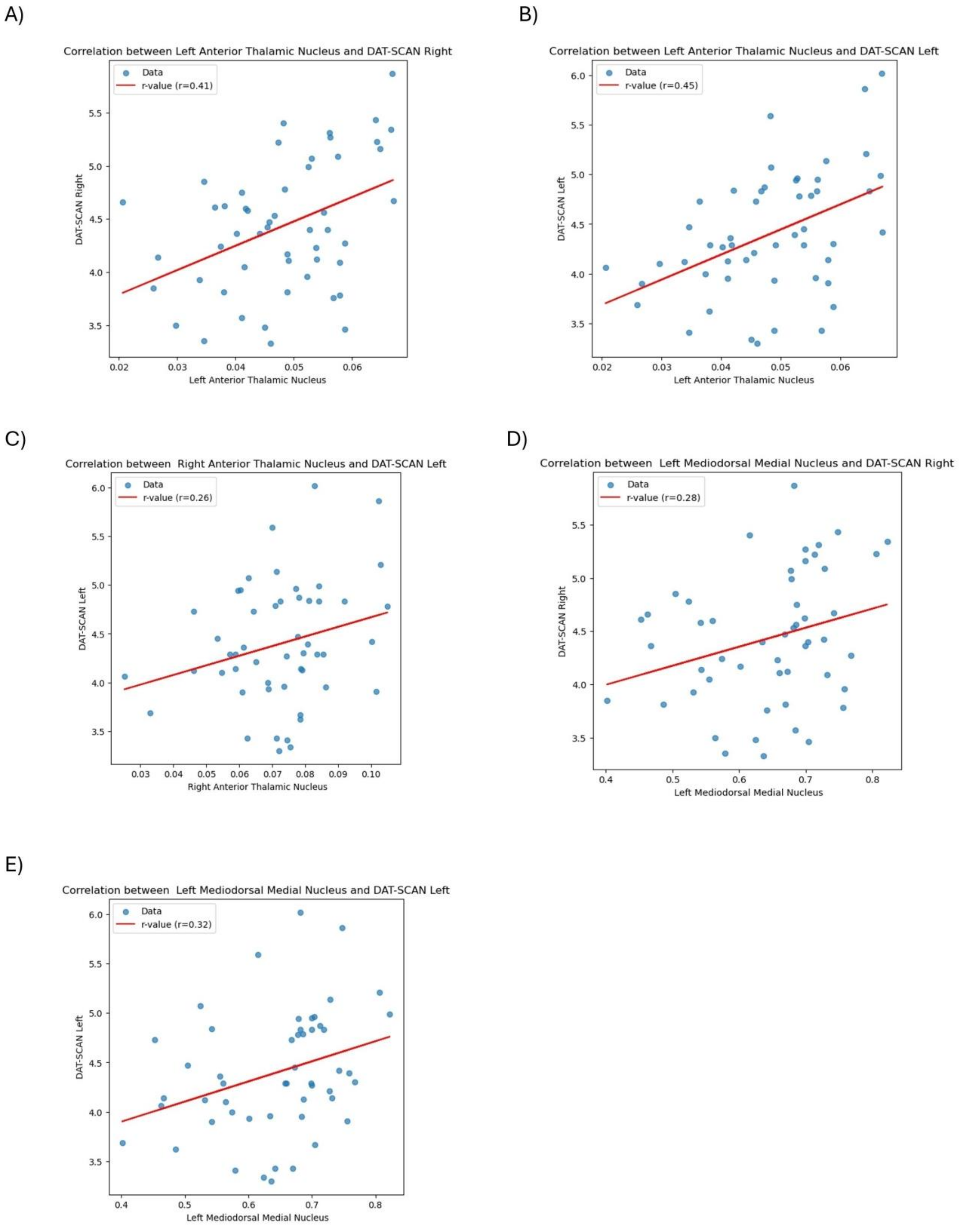
A)
Correlation between Left Anterior Thalamic Nucleus and DAT-SCAN Right
Data
r-value (r=0.41)
DAT-SCAN Right
Left Anterior Thalamic Nucleus
B)
Correlation between Left Anterior Thalamic Nucleus and DAT-SCAN Left
Data
r-value (r=0.45)
DAT-SCAN Left
Left Anterior Thalamic Nucleus
C)
Correlation between Right Anterior Thalamic Nucleus and DAT-SCAN Left
Data
r-value (r=0.26)
DAT-SCAN Left
Right Anterior Thalamic Nucleus
D)
Correlation between Left Mediodorsal Medial Nucleus and DAT-SCAN Right
Data
r-value (r=0.28)
DAT-SCAN Right
Left Mediodorsal Medial Nucleus
E)
Correlation between Left Mediodorsal Medial Nucleus and DAT-SCAN Left
Data
r-value (r=0.32)
DAT-SCAN Left
Left Mediodorsal Medial Nucleus
